# Machine learning models to predict the delivered positions of Elekta multileaf collimator leaves for volumetric modulated arc therapy

**DOI:** 10.1002/acm2.13667

**Published:** 2022-06-07

**Authors:** Sruthi Sivabhaskar, Ruiqi Li, Arkajyoti Roy, Neil Kirby, Mohamad Fakhreddine, Nikos Papanikolaou

**Affiliations:** ^1^ Department of Radiation Oncology The University of Texas Health Science Center at San Antonio San Antonio Texas USA; ^2^ Department of Management Science and Statistics The University of Texas at San Antonio San Antonio Texas USA

**Keywords:** Elekta, log files, machine learning, MLC positional deviations, VMAT

## Abstract

**Purpose:**

Accurate positioning of multileaf collimator (MLC) leaves during volumetric modulated arc therapy (VMAT) is essential for accurate treatment delivery. We developed a linear regression, support vector machine, random forest, extreme gradient boosting (XGBoost), and an artificial neural network (ANN) for predicting the delivered leaf positions for VMAT plans.

**Methods:**

For this study, 160 MLC log files from 80 VMAT plans were obtained from a single institution treated on 3 Elekta Versa HD linear accelerators. The gravity vector, *X*1 and *X*2 jaw positions, leaf gap, leaf position, leaf velocity, and leaf acceleration were extracted and used as model inputs. The models were trained using 70% of the log files and tested on the remaining 30%. Mean absolute error (MAE), root mean square error (RMSE), the coefficient of determination *R* ^2^, and fitted line plots showing the relationship between delivered and predicted leaf positions were used to evaluate model performance.

**Results:**

The models achieved the following errors: linear regression (MAE = 0.158 mm, RMSE = 0.225 mm), support vector machine (MAE = 0.141 mm, RMSE = 0.199 mm), random forest (MAE = 0.161 mm, RMSE = 0.229 mm), XGBoost (MAE = 0.185 mm, RMSE = 0.273 mm), and ANN (MAE = 0.361 mm, RMSE = 0.521 mm). A significant correlation between a plan's gamma passing rate (GPR) and the prediction errors of linear regression, support vector machine, and random forest is seen (*p* < 0.045).

**Conclusions:**

We examined various models to predict the delivered MLC positions for VMAT plans treated with Elekta linacs. Linear regression, support vector machine, random forest, and XGBoost achieved lower errors than ANN. Models that can accurately predict the individual leaf positions during treatment can help identify leaves that are deviating from the planned position, which can improve a plan's GPR.

## 1 INTRODUCTION

Over the past few decades, radiation treatment delivery techniques have been improving. The introductions of intensity‐modulated radiation therapy (IMRT) and volumetric modulated arc therapy (VMAT) have provided a more conformal dose coverage to the target volumes while sparing the normal tissue and nearby organs at risk (OARs).^1^, ^2^ VMAT provides a conformal dose to the targets and OARs through the modulation of the beam. Beam modulation combined with the modulation of the multileaf collimator (MLC) positions, dose rate, and gantry rotation speed allows for a faster treatment delivery. As VMAT is a highly modulated technique, and due to the complexity of VMAT planning and delivery, there is a higher chance for potential discrepancies between the planned and delivered dose distributions.^3^ Nithiyanantham et al.^4^ reported that an MLC positional error beyond ±0.3 mm can lead to significant differences in dose distribution and an MLC error of ±0.5 mm resulted in a dose deviation of more than 3% for VMAT plans delivered using an Elekta linear accelerator (linac). Therefore, this calls for performing patient‐specific plan quality assurance (QA) and dosimetric verification prior to the delivery of VMAT to ensure a safe and accurate treatment.^5^

During VMAT, the MLC leaves are in motion throughout the treatment when the beam is on. Therefore, MLC positional accuracy is crucial to prevent radiation toxicities to normal tissues and an underdosage to the tumor. A couple of factors that can lead to MLC positional deviations are the gantry angle and leaf velocity. Gantry angle can cause deviations in leaf positions due to the effect of gravity. A study by Ju et al.^6^ observed a maximum error in leaf position at 90° as the gantry rotated clockwise. As the gantry angle reached 180°, these errors decreased. A similar trend in the error was observed when the gantry was rotated anticlockwise. Another factor that can affect MLC leaf positions is the leaf velocity. VMAT plans often require the MLC leaves to move at a higher velocity so that the leaves can reach their next planned position in time. However, interleaf friction can affect leaf velocity,^7^ causing the leaves to move slower than their intended velocity and not reaching their next planned position fast enough. Wijesooriya et al.^8^ and Ling et al.^9^ reported higher MLC leaf positional errors in leaves moving at a faster velocity.

VMAT and IMRT are complex treatment delivery techniques that can introduce potential errors during treatment delivery, so pretreatment patient‐specific QA is performed prior to treatment delivery to identify discrepancies between the planned and delivered treatment. In highly modulated plans, the accuracy of the MLC leaf positioning is crucial.^10^ Therefore, ML models that can accurately predict the MLC leaf positions can be used to identify IMRT and VMAT plans that will potentially fail QA due to inaccurate MLC leaf positioning. As a result, the treatment planner can reduce the plan complexity ahead of time, thereby reducing chances for QA failures by predicting the delivered leaf positions.

Many studies have explored the application of conventional machine learning (ML) algorithms and ML‐based neural networks to successfully predict Varian MLC leaf positional errors. According to literature, conventional ML algorithms are outperformed by ML‐based neural networks.^11^ Carlson et al.^12^ were the first to develop ML models to predict the MLC positional deviations by using DynaLog files. They developed a linear regression, random forest, and cubist ML algorithms to predict the MLC positional deviations during VMAT delivery using DynaLog files and to examine the impact of these deviations on QA and dosimetry. The results from the study showed that the cubist model outperformed the other models in accurately predicting the MLC positional errors. Osman et al.^5^ developed an ML method based on a feedforward artificial neural network (ANN) to predict the individual MLC leaf positional deviations during the dynamic IMRT delivery priori using data from Varian DynaLog files. The results from this study showed that the ANN model outperformed the accuracy of previous ML models in literature, and the model could be applied to dose calculations and optimization to improve the gamma passing rate (GPR) for patient‐specific IMRT QA. The ANN model developed by Osman et al.^5^ outperforms the models developed by Carlson et al.^12^ in predicting MLC positional errors. Chuang et al.^13^ developed several regression models, such as simple/multiple linear regression, decision tree, bagged tree, and boosted tree models to predict MLC discrepancies during IMRT and VMAT based on MLC motion parameters from trajectory log files from Varian linac.

The use of log files for pretreatment patient‐specific QA has increased in the recent years. An advantage of the log file–based QA over the traditional measurement‐based QA is that log files contain the delivered machine parameters and MLC leaf positions. Incorporating the machine parameters into the treatment planning system to recompute the dose can show the deviations between the planned and delivered dose. Because MLC leaf positioning is the largest source of error during treatment delivery,^10^ ML models can be incorporated into the pretreatment patient‐specific QA workflow for predicting the delivered MLC leaf positions at the time of treatment planning. These predictions can be used to compute the delivered dose, which can be compared to the planned dose for plan verification.

Although several studies have examined conventional ML algorithms and neural networks to predict Varian MLC positional deviations, this study is the first application of ML techniques to predict the delivered Elekta MLC leaf positions. There are differences between the motion control system of an Elekta MLC and a Varian MLC. In Varian, the MLC is placed as a tertiary system below the upper and lower jaws. This design places the MLC closer to the patient than in an Elekta linac.^14^ Having the MLC below the jaws adds extra bulk to the system, because beam divergence requires a larger system to cover the same field size. Furthermore, placing the MLC farther from the X‐ray source requires an increase in the leaf length and the distance traveled by the leaves from one side of the field to the other.^15^ As an effort to reduce the distance the leaves travel across the field, the leaves travel on a carriage. Varian MLC leaves have a higher transmission but lower interleaf leakage than Elekta.^16^ In Elekta, the MLC replaces the upper jaw, and the leaves move only in the *y*‐direction.^14^ The closer placement of the MLC to the X‐ray source reduces the distance the leaves must travel, thus allowing for a shorter leaf length and an overall reduction in the size of the system.^14^ The disadvantage of this design is the smaller leaf width, which calls for a tighter tolerance on leaf positioning and leaf travel.^14^ Compared to Varian, Elekta MLC has lower transmission but higher interleaf leakage.^16^ This study focuses on examining different conventional ML models and an ML‐based neural network to predict the delivered positions of individual MLC leaves for VMAT treatment plans delivered using an Elekta linac.

## 2 MATERIALS AND METHODS

### 2.1 Elekta MLC log files

The Elekta Agility MLC system consists of 160 individual leaves (80 leaves in the *X*1 bank and 80 leaves in the *X*2 bank). Unlike the Varian system that uses potentiometers and encoders for the MLC position verification,^2^, ^17^ the Elekta system uses optical technology.^2^ The Elekta Agility uses a Rubicon optical positioning system, which allows for accurate positioning of MLC leaves.^2^

During the treatment, the Elekta system records the mechanical information and delivery parameters of the linac every 40 ms. The Elekta log files contain information about the control points, linac state, dose rate, delivered dose, wedge information, gantry angle, collimator angle, *X*1 jaw position, *X*2 jaw position, individual leaf positions, table positions, and the errors associated with each of these parameters.^2^ The retrieved Elekta log files are in a binary format with a .trf (treatment record file) extension and needed to be converted to a readable ASCII format. To do this, an in‐house MATLAB algorithm was previously written.^2^

### 2.2 Predictive planning parameters

For this study, 160 MLC log files from 80 VMAT plans were retrospectively acquired from a single institution treated on 3 Elekta Versa HD linacs. All the plans were generated in the Pinnacle treatment planning system. The treatment sites of the VMAT plans included are head‐and‐neck, pelvis, prostate, brain, lung, and abdomen. All plans were treated with two arcs using beam energy of either 6 or 10 MV. For the three linacs, we found limited differences in treatment delivery parameters during commissioning and QA; therefore, a generalized model was built to predict the behavior of the three linacs.

The planned parameters considered to be predictive in determining the delivered MLC leaf positions were extracted or calculated from the log files. Based on what has been reported in literature, the following parameters (Table 1) were extracted from the log files: gantry angle,^6^ collimator angle,^18^ *X*1 jaw position,^19^ *X*2 jaw position,^19^ and leaf position.^5^, ^12^ Gravity vector,^13^ leaf gap,^3^, ^5^ leaf velocity,^3^, ^5^, ^8^, ^9^, ^12^, ^20^ and leaf acceleration^3^, ^9^ were calculated from the extracted parameters according to the formulas shown in the second column of Table 1. The seven planned parameters shown in Table 1 are used as inputs to the multiple linear regression, support vector machine, random forest, extreme gradient boosting (XGBoost), and ANN, which are discussed separately in the following section.

**TABLE 1:** Planned input parameters to predict the delivered multileaf collimator (MLC) leaf positions

Planning parameter	Formula
Gravity vector	cos(collimator angle) × sin(gantry angle)
*X*1 jaw position	–
*X*2 jaw position	–
Leaf position	–
Leaf gap	abs(leaf(bankX1)−leaf(bankX2))
Leaf velocity	$\frac{p_{t}-\;p_{t-1}}{\mathrm{time}\;(0.040\;s)}$
Leaf acceleration	$\frac{v_{t}-\;v_{t-1}}{\mathrm{time}\;(0.040\;s)}$

*Note*: The current leaf position is represented by *p_t_*, and the leaf position at the previous timepoint is represented by *p_t−1_*. The current leaf velocity is represented by *v_t_*, and the leaf velocity at the previous timepoint is represented by *v_t−1_*.

### 2.3 Multiple linear regression

Linear regression is a statistical method that quantifies the relationship between the independent and dependent variables.^21^ Multiple linear regression estimates the linear effect that one independent variable has on the dependent variable after adjusting for the effects of confounders, which are variables that influence both the dependent and independent variables.^22^ This effect is described by the regression coefficient.^23^ The regression coefficient denotes the change in the dependent variable per unit change in the independent variable^24^ and is estimated by least squares. A multiple linear regression model was developed to evaluate the relationship between the planned parameters and the delivered MLC leaf positions, as linear regression coefficients, discussed in Section 3, are more intuitively understandable. The features were scaled for this model.

### 2.4 Support vector machine

Support vector machine uses a kernel, such as linear, polynomial, Gaussian, radial basis function (RBF), or sigmoid, to transform the input data for processing. The kernel finds a hyperplane containing the maximum number of data points. An *ԑ*‐insensitive tube surrounds the hyperplane, where *ԑ* specifies the margin for error tolerance. The symmetric loss function penalizes data points outside the *ԑ*‐insensitive tube, whereas the data points inside the tube are not. Essentially, this model tries to find the narrowest tube that minimizes the prediction errors.^25^ The regularization parameter *C* specifies the amount of misclassification to be avoided. Support vector machine was selected due to its ability to not over‐fit, handle nonlinear data, not rely on any distributional assumptions, and stability, because a small change in the data does not produce significant changes to the hyperplane. However, a disadvantage is that it cannot handle unbalanced data well.

Hyperparameter tuning was done using sixfold cross‐validation, and the metrics used to validate the model performance were the mean absolute error (MAE), root mean square error (RMSE), and the coefficient of determination *R* ^2^. During grid search, the hyperparameter values examined were the following: kernel: [linear, RBF], *C*: [0.40, 0.60, 0.80], and *ԑ*: [0.01, 0.05, 0.10]. The final values selected for the model were kernel = linear, *C* = 0.40, and *ԑ* = 0.01. The features were scaled for this model.

### 2.5 Random forest

Random forest is an ensemble learning algorithm, where results from multiple predictions are averaged to obtain the final prediction that is more accurate and stable. When building the random forest, each decision tree is trained parallelly, so there is no interaction between them,^26^, ^27^, ^28^ and bagging is used to construct each tree, where a randomly chosen subset of features and training data are used. This nonparametric model was selected because it does not rely on any distributional assumptions, handles large numbers of input variables and imbalanced data well, reduces overfitting by bagging, and reduces correlation between different trees by the random sampling of coefficients at each node. Random forest's feature importance, discussed in Section 3, provides information on how important each of the features is in making the predictions.

Random forest's hyperparameters control the structure of each decision tree, the forest, and its level of randomness.^29^ Maximum depth is the number of nodes that is allowed from the root to the farthest leaf in the tree, maximum features are the maximum number of features considered for splitting a node, minimum sample of splits is the minimum number of data points allowed in a node before splitting the node, and minimum samples of leaf are the minimum number of data points allowed in a leaf node. Hyperparameter tuning was done with sixfold cross‐validation, and the hyperparameter values examined were the following: number of trees: [50, 100, 150, 200], maximum depth: [15, 20, 25, 30], maximum features: [none, auto], minimum sample of splits: [4, 6, 8, 10], and minimum samples of leaf: [4, 6, 8, 10]. The final values selected were the number of trees = 200, maximum depth = 30, maximum features = none, minimum sample of splits = 8, and minimum samples of leaf = 4.

### 2.6 XGBoost

XGBoost is a gradient‐boosted regression tree algorithm that applies the principles of gradient descent and gradient boosting. Gradient descent is an optimization algorithm that is used to minimize the cost function, which measures how close the predictions are to the true values. Gradient descent runs the model with initial weights, and then updates the weights through several iterations, thus minimizing the cost function. The model's weights affect how close the predictions are to the true values. The trees are built sequentially, so each tree learns from and reduces the errors made by the previous trees. XGBoost was selected because this algorithm is an ensemble of weak learners combined to produce a single strong learner,^30^ which is an advantage of XGBoost. XGBoost's feature importance, discussed in Section 3, provides information on how important each of the features is in making the predictions.

The learning rate controls the rate at which the model learns from the patterns in the data. After a tree is added to the model, the learning rate shrinks the weights to make the model more robust and conservative. Shrinkage reduces the influence of the individual tree and allows for future trees to improve the model. Although controlling for the learning rate can improve the accuracy of the prediction, it can also increase the time for training the model. The minimum child weight is the minimum weight that is required before creating a new node in the tree. Hyperparameter tuning was done with sixfold cross‐validation, and the hyperparameter values examined were the following: learning rate: [0.10, 0.15, 0.20, 0.25, 0.30], maximum depth: [10, 15, 20, 25, 30], and minimum child weight: [2, 4, 6, 8, 10]. The final values were learning rate = 0.20, maximum depth = 20, and minimum child weight = 10.

### 2.7 Artificial neural network

ANN imitates the biological neural network of the brain and processes information the way the brain does. The neuron is the basic structure of the ANN, and the inputs to a neuron are weighted by first multiplying the input value by individual weight. These weighted inputs and biases are summed and passed through an activation function, which processes this information and passes it via the output. ANN consists of input, hidden, and output layers. Between the input and output layers, are several hidden layers. The neurons in the hidden layers are interconnected and receive information from all the neurons in the layer above them.^31^ The number of hidden layers, the number of neurons in each layer, and the activation function for each layer are modified to tune the ANN model. Several studies have studied neural networks for predicting MLC errors^5^ and for detecting errors in patient‐specific QA for VMAT^32^ and IMRT.^33^ The accuracy of the ANN developed by Osman et al.^5^ for predicting MLC positional errors for IMRT delivered on Varian linac has led us to study the accuracy of ANN in predicting the positional deviations for VMAT delivered on Elekta linac.

The activation function of each layer impacts the performance of the neural network by determining how the sum of the weighted inputs is transformed into an output of a node in a layer. While building the model, different combinations of activation function, the number of hidden layers, and the number of neurons in each layer were tested. Sixfold cross‐validation was done to tune the model. The ANN was trained with 100 epochs and the Adam optimizer. During training, several different epoch values (50, 75, 100, 125, 150) were examined and the model performance was evaluated using MAE. For more than 100 epochs, the MAE on the validation set began increasing. As the model began converging at 100 epochs, the ANN model was trained with 100 epochs. The input layer consists of seven neurons and the ReLU activation function. All 4 hidden layers consist of 28 neurons, but the first 3 layers have the ReLU activation function, and the last layer has a linear activation function. The output layer consists of one neuron and the linear activation function. The features were scaled for this model.

### 2.8 Model training, validation, and testing

**FIGURE 1:**
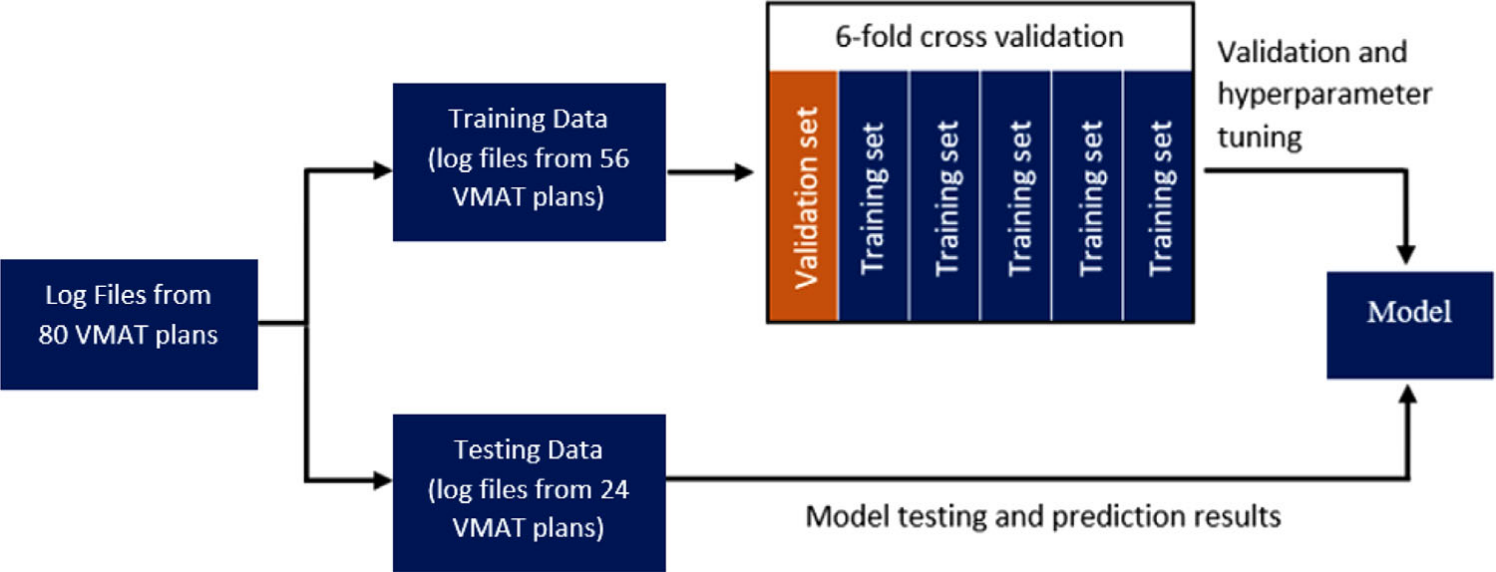
Methodology for developing the machine learning models

The models were developed in Python version 3.8.8. The scikit‐learn was used to build the linear regression, support vector machine, random forest, and XGBoost, and the TensorFlow was used to build the ANN. Figure 1 shows the methodology for building the models. For each of the algorithms in this study, an individual model for each leaf was built to predict the delivered positions for that leaf. For training, log files from 56 VMAT plans were used. The planned parameters and the delivered leaf positions were extracted from the log files, and the planned parameters were used as the model input and the delivered leaf position was the model target. To validate and tune the model's hyperparameters, sixfold cross‐validation was performed, where the training data is split into six different sets. Five of the sets are used to train the model, and the remaining set is used to validate the model's performance using MAE, RMSE, and the coefficient of determination *R* ^2^. This process is repeated six times so that the model is validated on each set. The MAE, RMSE, and *R* ^2^ achieved on the six validation sets were averaged to obtain a final result. The hyperparameters that gave the best results on the validation set were used as the final model.The remaining log files from the 24 VMAT plans were used to test the final model. The performances of the models on all the MLC leaves were evaluated using the Kruskal–Wallis test and the post hoc Dunn test. Essentially, the trained models can predict what the delivered MLC positions will be in VMAT plans before the treatment delivery by inputting the planned parameters.

## 3 RESULTS

### 3.1 MLC leaf positional deviations

**FIGURE 2:**
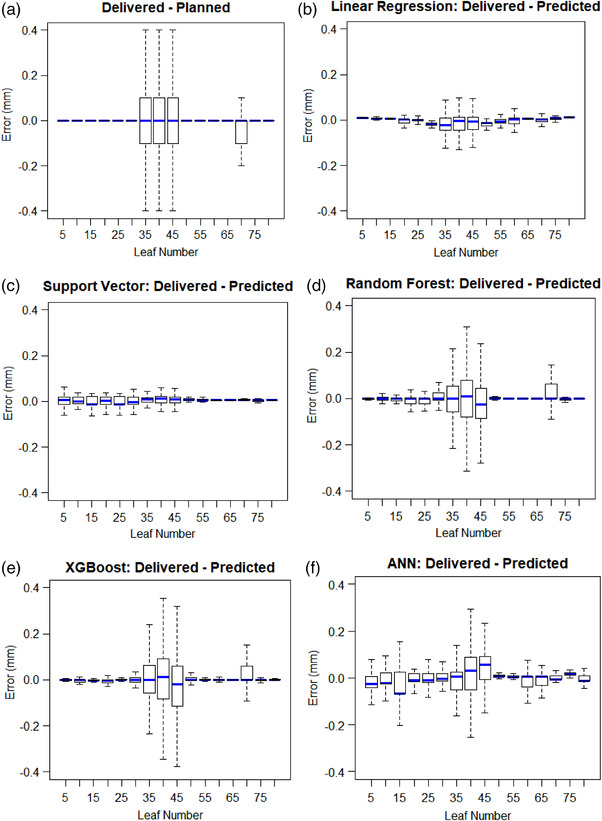
Multileaf collimator (MLC) deviations between the delivered and (a) planned positions and deviations between the delivered and predicted positions of the (b) linear regression, (c) support vector, (d) random forest, (e) extreme gradient boosting (XGBoost), and (f) artificial neural network (ANN) models

The positional deviations of 16 MLC leaves from *X*1 bank from one test plan are shown in the boxplots in Figure 2. These leaves were chosen to show how the positional deviations vary throughout all the leaves in the *X*1 bank. From Figure 2a, the positional errors (delivered–planned) are higher for the middle leaves, as the leaves in the middle of the field undergo more motion, where they might be required to travel larger distances at a faster speed during treatment than the leaves in the field edge. The leaves in the field edge are usually stationary or undergo less motion than the leaves in the field center. Therefore, more deviations in the leaf positions are seen for the leaves in the field center.

The boxplots in Figure 2b–f show the errors (delivered–predicted) of the linear regression, support vector machine, random forest, XGBoost, and ANN, respectively. Linear regression's (Figure 2b) and support vector machine's (Figure 2c) magnitude of errors are much lower for the central leaves when compared to the magnitude of errors from Figure 2a. The errors from the models are either comparable or slightly higher for the outer leaves. Random forest (Figure 2d), XGBoost (Figure 2e), and ANN (Figure 2f) also show a lower magnitude of errors than the errors from Figure 2a for the central leaves, but these errors are relatively higher than the errors from linear regression and support vector machine. However, the errors from the models are either comparable or slightly higher for the outer leaves.

### 3.2 Model performance

The MAE, RMSE, and *R* ^2^ values for the training, validation, and testing datasets along with the standard deviations in the errors achieved by the five models on the VMAT plans from the testing dataset are shown in Table 2. To see if there are statistically significant differences in the MAE and RMSE achieved by the models, the nonparametric Kruskal–Wallis test was performed. The Kruskal–Wallis test was chosen instead of the ANOVA, because the errors were non‐normal. Results from the Kruskal–Wallis test indicate that there is a significant difference in the MAE (*p* = 5.428 × 10^−4^) and the RMSE (*p* = 5.243 × 10^−4^) achieved by the models. This shows that at least one of the model's MAE and RMSE are statistically different from the others. To see which model is statistically different from the others, the post hoc nonparametric pairwise comparison of the models using the Dunn test was performed. The result of the post hoc Dunn test on the MAE and RMSE is shown in Table 3. A pairwise comparison of ANN's MAE and RMSE shows statistically significant difference in the MAEs and RMSEs achieved by linear regression (*p* = 0.016, *p* = 0.022), support vector (*p* = 3.765 × 10^−4^, *p* = 3.290 × 10^−4^), and random forest (*p* = 0.020, *p* = 0.029). ANN's MAE and RMSE are not significantly different from XGBoost's MAE (*p* = 0.358) and RMSE (*p* = 0.517). The remaining pairwise comparisons of the MAE achieved by the models show nonsignificant differences.

**TABLE 2:** Model performance in predicting delivered leaf position on the training, validation, and testing datasets

	Training			Validation			Testing					
Model	MAE (mm)	RMSE (mm)	*R* ^2^	MAE (mm)	RMSE (mm)	*R* ^2^	MAE (mm)	SD (mm)	RMSE (mm)	SD (mm)	*R* ^2^	SD
Linear regression	0.188	0.307	0.999	0.192	0.310	0.995	0.158	0.054	0.225	0.068	0.916	0.096
Support vector	0.214	0.337	0.999	0.222	0.376	0.995	0.141	0.048	0.199	0.065	0.928	0.097
Random forest	0.111	0.204	0.999	0.250	0.716	0.992	0.161	0.050	0.229	0.069	0.926	0.096
XGBoost	0.072	0.117	0.999	0.272	0.761	0.989	0.185	0.062	0.273	0.107	0.906	0.126
ANN	0.224	0.351	0.999	0.241	0.459	0.996	0.361	0.240	0.521	0.351	0.914	0.096

*Note*: For the testing dataset, the average mean absolute error (MAE), root mean square error (RMSE), *R* ^2^, achieved by the models across all plans in the testing dataset, along with the standard deviation (SD) are reported.

Abbreviations: ANN, artificial neural network; MAE, mean absolute error; RMSE, root mean square error; SD, standard deviation; XGBoost, extreme gradient boosting.

**TABLE 3:** Results of post hoc Dunn test for the mean absolute error (MAE) and root mean square error (RMSE)

Comparison		MAE		RMSE	
Model 1	Model 2	*Z*‐Test statistic	*p*‐Value	*Z*‐Test statistic	*p*‐Value
ANN	Linear regression	3.126	0.016*	3.025	0.022*
ANN	Support vector	4.121	3.765 × 10^−4^ ***	4.152	3.290 × 10^−4^ ***
ANN	Random forest	3.029	0.020*	2.914	0.029*
ANN	XGBoost	1.884	0.358	1.716	0.517
Linear regression	Random forest	−0.097	0.922	−0.111	0.912
Linear regression	Support vector	0.995	0.639	1.128	0.519
Linear regression	XGBoost	−1.243	1.000	−1.309	0.953
Random forest	Support vector	1.092	0.824	1.238	0.863
Random forest	XGBoost	−1.145	1.000	−1.198	0.692
Support vector	XGBoost	−2.238	0.177	−2.437	0.104

Abbreviations: ANN, artificial neural network; MAE, mean absolute error; RMSE, root mean square error; XGBoost, extreme gradient boosting.

* *p* < 0.05;

** *p* < 0.01;

*** *p* < 0.001.

### 3.3 Fitted line plots

**FIGURE 3:**
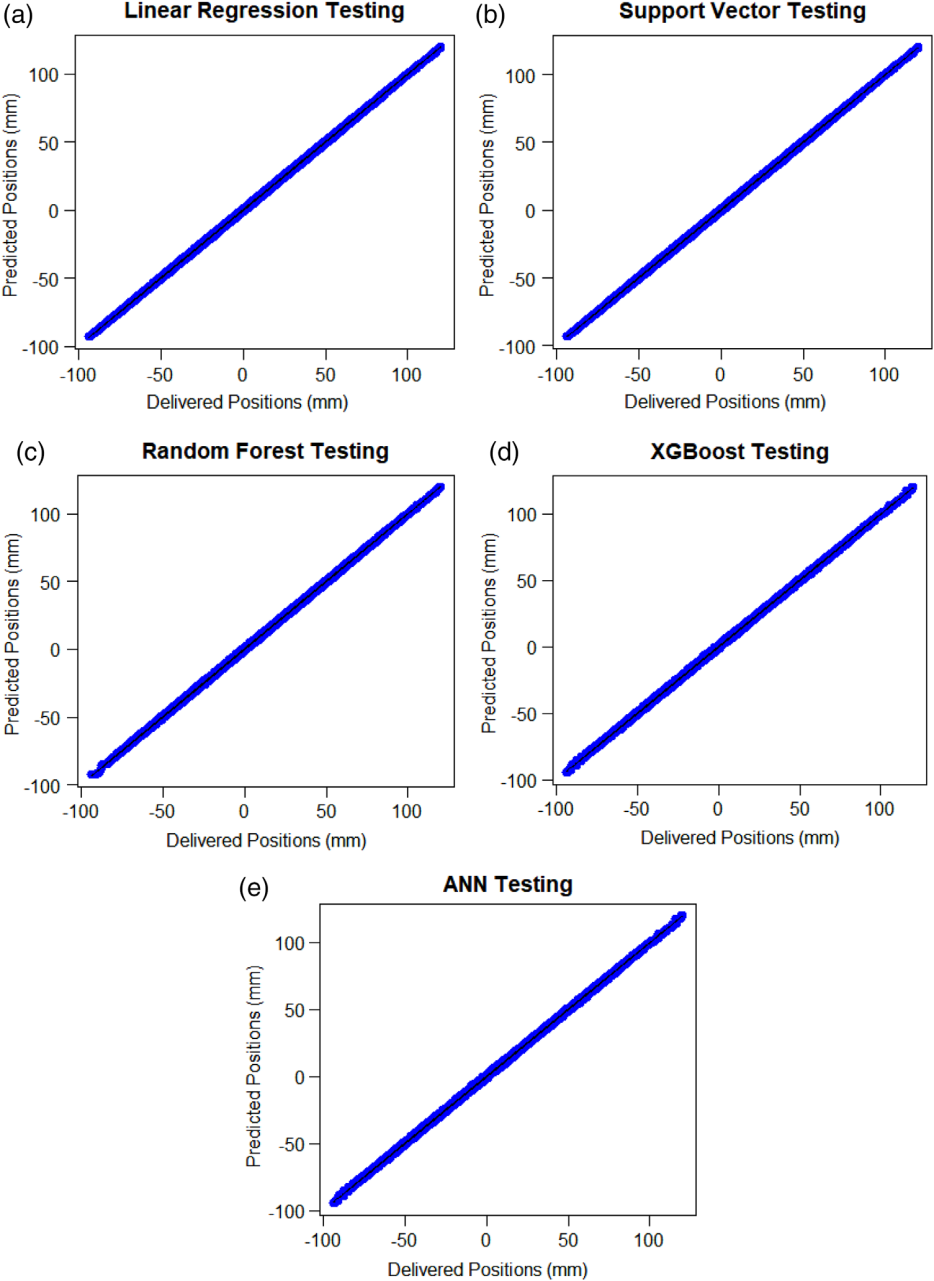
Fitted‐line plots showing the relationship between the delivered and predicted positions for a single volumetric modulated arc therapy (VMAT) plan from the testing data set for (a) linear regression, (b) support vector, (c) random forest, (d) extreme gradient boosting (XGBoost), and (e) artificial neural network (ANN)

The fitted line plots in Figure 3a–e show the relationship between the predicted and delivered leaf positions during testing of the linear regression, support vector machine, random forest, XGBoost, and ANN, respectively. The dashed line denotes a perfect agreement between the predicted and delivered positions. Ideally, the points should be close to the dashed line. All five models show a good fit between the delivered and predicted MLC leaf positions during testing.

### 3.4 Linear regression coefficients

The regression coefficients represent the mean change in the dependent variable when the independent variable is given a one‐unit shift. The magnitude of the coefficient indicates the size of impact the independent variable has on the independent variable, and the significance of the variable is determined by the *p*‐value. The significance of the independent variable was evaluated with a significance level of 0.05.

The regression coefficients and the *p*‐values for each feature are shown in Table 4. The significant features are the leaf position (*p* < 2.00 × 10^−16^), leaf velocity (*p* = 5.510 × 10^−5^), leaf acceleration (*p* < 2.00 × 10^−16^), *X*1 jaw position (*p* = 0.009), *X*2 jaw positions (*p* = 0.002), and leaf gap (*p* = 0.029). This implies that any changes in these features are associated with changes in the delivered positions. The gravity vector has the least significance (*p* = 0.393), meaning any change in this feature is not associated with changes in the delivered leaf positions.

**TABLE 4:** Linear regression coefficients and *p*‐values for each feature

Feature	Regression coefficient	*p*‐Value
Gravity vector	−2.49 × 10^−3^	0.393
*X*1 jaw position	−3.57 × 10^−4^	0.009**
*X*2 jaw position	6.817 × 10^−4^	0.002**
Leaf gap	−7.246 × 10^−6^	0.029*
Leaf position	0.998	<2.00 × 10^−16^ ***
Leaf velocity	−2.417 × 10^−4^	5.510 × 10^−5^ ***
Leaf acceleration	1.891 × 10^−4^	<2.00 × 10^−16^ ***

* *p* < 0.05;

** *p* < 0.01;

*** *p* < 0.001.

To ensure that the results from the linear regression analysis are reliable, multicollinearity must not exist in the data. Multicollinearity is when the independent variables are correlated to each other. As regression coefficients denote the mean change in the dependent variable for each unit change in the independent variable, although the other independent variables are held constant, if two variables are correlated, a change in one variable leads to a change in the other variable. Therefore, this can be problematic when fitting the model and interpreting the regression coefficients. Multicollinearity was checked by computing the correlation matrix and the variance inflation factor (VIF).

VIF estimates of how much the regression coefficient's variance is inflated due to the presence of multicollinearity in the regression model. A VIF of 1 means there is no correlation, a VIF between 1 and 5 means there is moderate correlation, and a VIF greater than 5 means there is high correlation. The VIFs of each feature were below the threshold value of 5, so there is no multicollinearity. The strongest correlation is seen between *X*1 jaw position and leaf gap, with a coefficient of 0.41, which is below the threshold of 0.70 for multicollinearity. Therefore, multicollinearity does not exist in the data.

### 3.5 Random forest and XGBoost feature importance

**FIGURE 4:**
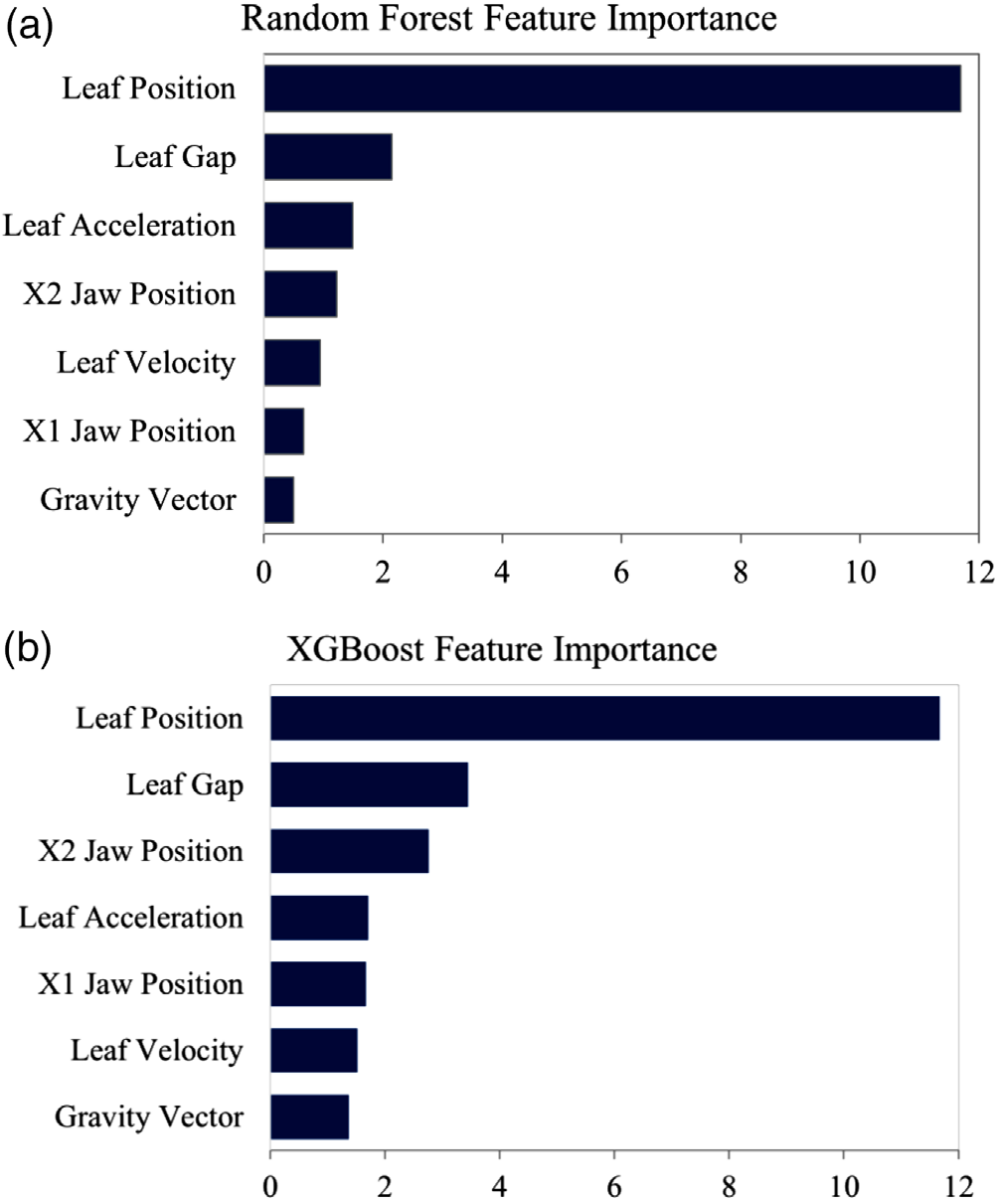
Feature importance of (a) random forest and (b) extreme gradient boosting (XGBoost) models

Shown in Figure 4 are the log‐transformed feature importance of the random forest (Figure 4a) and XGBoost (Figure 4b). Permutation feature importance is the decrease in the model score when a single feature is randomly shuffled.^25^ In permutation feature importance, the relationship between a particular feature and the target variable is broken. Therefore, a decrease in the model score indicates the extent to which the model depends on that feature. The feature importance provides information on which features should be selected as model inputs to reduce overfitting. Random forest and XGBoost ranked leaf position as the most important feature, which matches the linear regression's results. Leaf gap was the second most important feature in the random forest and XGBoost models. Both models ranked gravity vector as the least important feature. The results from linear regression indicate that gravity vector has less significance in predicting the delivered positions, which matches the feature importance of random forest and XGBoost.

### 3.6 Positional deviations and gamma passing rates

For the 24 plans from the testing dataset, QA was performed with gamma criteria of 3%/2 mm. The GPRs ranged from 90.7% to 99.7%. To evaluate the impact of the leaf positional errors on the GPRs, the correlation between positional errors (planned–delivered) and GPR was evaluated for the plans. As the average errors increased, the GPR of the plan decreased. The result from a Pearson correlation test between the errors (*r* = −0.456, *p* = 0.024) shows a significant correlation between the errors and GPR at a significance level of 0.05.

To examine the impact of the model prediction errors on the GPRs, a Pearson correlation test between prediction errors (planned–predicted) and GPR was performed. Linear regression (*r* = −0.425, *p* = 0.039), support vector (*r* = −0.415, *p* = 0.043), and random forest (*r* = −0.481, *p* = 0.020) show a significant correlation between the errors and GPR. As the deviations from the planned and predicted positions increase, the GPR decreases. However, XGBoost (*r* = −0.290, *p* = 0.228) and ANN (*r* = −0.325, *p* = 0.107) do not show a significant correlation between the errors and GPR. Therefore, the deviations between the planned and predicted positions by linear regression, support vector, and random forest seem to be a good indicator of the GPR of a plan.

## 4 DISCUSSION

Deviations between planned and delivered MLC leaf positions can lead to significant errors in dose distribution during IMRT and VMAT. Therefore, accurate MLC leaf positioning is crucial during these treatments. In this study, we developed ML‐based linear regression, support vector machine, random forest, XGBoost, and ANN models for predicting the delivered positions of individual MLC leaves for VMAT treatments using MLC log files data from a single institution. Various studies in literature have reported the impact of MLC positional deviations on the delivered dose distribution. Nithiyanantham et al.^4^ reported an MLC positional error of more than ±0.3 mm can have a significant impact on VMAT dose distribution and an error of ±0.5 mm can cause 3% dose error. Oliver et al.^34^ examined MLC errors in VMAT plans and recommended the errors to be within ±0.6 mm to keep the target dose accuracy to within ±2%.

The results from this study are compared with the studies by Carlson et al.^12^ and Osman et al.,^5^ which examined machine and deep learning models for predicting MLC positional errors on a Varian linac. Carlson et al.^12^ reported MAE for the random forest from the three institutions are 0.124, 0.089, and 0.165 mm, and their reported RMSE are 0.244, 0.200, and 0.323 mm. The MAE and RMSE achieved by our random forest are comparable to the errors reported by Carlson et al.^12^ Their reported MAEs for the linear regression from the three institutions are 0.134, 0.086, and 0.162 mm, and their reported RMSEs are 0.253, 0.193, and 0.323 mm from the three institutions. The MAE and RMSE achieved by our linear regression are comparable to their reported errors. Similarly, our support vector machine's and XGBoost's MAE and RMSE are also comparable. Osman et al.^5^ reported MAE and RMSE for the ANN are 0.006 and 0.0097 mm, respectively, which is much lower than the errors achieved by our ANN. A possible reason for our ANN achieving higher errors than the ANN built by Osman et al.^5^ is the limited number of input features used in this study. Osman et al.^5^ included more input features and leaf motion parameters that were not included for training the models in this study, namely, the leaf's current, previous, and next position and whether the leaf was starting, resting, or accelerating. Another possible reason is the hyperparameters used for tuning and validating the models. Although our ANN achieved higher errors than the errors reported by Osman et al.,^5^ this is the first study to investigate ML models for predicting leaf positions of an Elekta MLC system.

Although our linear regression, support vector machine, random forest, and XGBoost achieved lower errors than ANN, when evaluating the models’ prediction errors with the GPR, a significant correlation is seen between the prediction errors of linear regression, support vector machine, and random forest with the GPR. The correlation between the GPR and the prediction errors of ANN and XGBoost is nonsignificant. This shows that the deviations between the planned and predicted leaf position by linear regression, support vector, and random forest seem to be a good indication of a plan's GPR. The feature importance of random forest and XGBoost are in agreement with linear regression results. Random forest and XGBoost ranked leaf position and leaf gap as the top two important features and the gravity vector with relatively less importance. This matches the results from linear regression, which indicates that the significant features are the leaf position, leaf velocity, leaf acceleration, jaw positions, and leaf gap, whereas the gravity vector had no significance in predicting the delivered leaf positions.

Due to the complexity of VMAT techniques, many factors can lead to the introduction of errors during treatment, thus reducing the accuracy in VMAT delivery. Several studies have investigated the dosimetric effects of systematic shifts in MLC leaf positions and leaf gap on the dose distribution for IMRT^35^, ^36^, ^37^ and VMAT.^33^, ^38^ Furthermore, a study by Park et al.^3^ reported a decrease in VMAT delivery accuracy as the leaf speed and acceleration increased. To ensure a safe and accurate delivery of VMAT, patient‐specific QA and dosimetric verification is performed prior to treatment delivery, which is often a time‐consuming process. Models that can predict what the delivered position of each individual leaf will be during treatment are advantageous, as they aid in identifying specific leaves that are deviating from the planned position, and thus leading to differences in the planned and delivered dose distribution. With the models from this study, one can identify individual leaves that are deviating by a large amount from the planned positions, either due to an increased leaf travel or other planned parameters that keep the leaf from reaching the planned position on time. Knowing this information during the treatment planning process allows one to correct this by reducing the complexity of the VMAT plan to improve the plan's QA outcome.

The use of MLC log files to evaluate the performance of the MLC and to detect positional errors has increased. However, when performing log file–based patient‐specific QA, it might be necessary to use an electronic portal imaging device to verify that the recorded MLC positions in the log files are the actual delivered MLC positions. This was not a part of this study, and further investigation is needed to verify this for the Elekta Agility collimator. A few other limitations of this study are that only the Elekta Agility MLC system was considered, and the log files data used to build the ML models were obtained retrospectively from a single institution. Therefore, using data from other treatment planning systems or different types of MLC systems, such as Varian, might lead to discrepancies in the predicted MLC leaf positions. This is mainly because the MLC control system of each linac differs from one another based on its design and placement. Another thing to consider when applying these models for a new patient is the different sampling times of the DICOM‐RT and the log files. The log files used in this study have a sampling time of 40 ms, which may not be the sampling time of the DICOM‐RT. Therefore, these differences in sampling times must be taken into account by synchronization.^12^

## 5 CONCLUSIONS

In this study, we developed ML‐based linear regression, support vector machine, random forest, XGBoost, and ANN models to predict the delivered positions of individual MLC leaves for VMAT treatment delivery using an Elekta linac. Based on the MAE, RMSE, and fitted line plots, linear regression and support vector machine show higher accuracy than random forest, XGBoost, and ANN models developed in this study. Having an accurate model for predicting the MLC positional deviations will be a useful tool. It allows the treatment planner to identify IMRT or VMAT plans that are most likely to fail QA ahead of time based on the predicted MLC positional errors, and thus reduce the number of plans that fail patient‐specific QA.

## CONFLICT OF INTEREST

The authors declare that there is no conflict of interest that could be perceived as prejudicing the impartiality of the research reported.

## AUTHOR CONTRIBUTIONS

All authors contributed substantially to the design of the study. Sruthi Sivabhaskar conducted the study and all authors contributed to the analysis and interpretation of the results. Sruthi Sivabhaskar drafted the manuscript and all authors provided critical feedback on revising the manuscript for important intellectual content and all authors gave their final approval of the version to be published.
